# Assessing causal relationships using genetic proxies for exposures: an introduction to Mendelian randomization

**DOI:** 10.1111/add.14038

**Published:** 2017-11-03

**Authors:** Srinivasa Vittal Katikireddi, Michael J. Green, Amy E. Taylor, George Davey Smith, Marcus R. Munafò

**Affiliations:** ^1^ MRC/CSO Social and Public Health Sciences Unit University of Glasgow Glasgow UK; ^2^ MRC Integrative Epidemiology Unit University of Bristol Bristol UK; ^3^ UK Centre for Tobacco and Alcohol Studies, School of Experimental Psychology University of Bristol Bristol UK

**Keywords:** Addictive behaviour, causality, econometric models, epidemiological methods, genetic epidemiology, Mendelian randomization analysis

## Abstract

**Background and aims:**

Studying the consequences of addictive behaviours is challenging, with understanding causal relationships from observational data being particularly difficult. For example, people who smoke or drink excessively are often systematically different from those who do not, are less likely to participate in research and may misreport their behaviours when they do. Furthermore, the direction of causation between an addictive behaviour and outcome may be unclear. Mendelian randomization (MR) offers potential solutions to these problems.

**Methods:**

We describe MR's principles and the criteria under which it is valid. We identify challenges and potential solutions in its application (illustrated using two applied examples) and describe methodological extensions in its application.

**Results:**

MR is subject to certain assumptions, and requires the availability of appropriate genetic data, large sample sizes and careful design and conduct. However, it has already been applied successfully to the addiction literature. The relationship between alcohol consumption (proxied by a variant in the *ADH1B* gene) and cardiovascular risk has been investigated, finding that alcohol consumption increases risk, with no evidence of a cardioprotective effect at moderate consumption levels. In addition, heaviness of smoking (proxied by a variant in the *CHRNA5‐A3‐B4* gene cluster) and risk of depression and schizophrenia have been investigated, with no evidence of a causal effect of smoking on depression but some evidence of a causal effect on schizophrenia.

**Conclusions:**

Mendelian randomization analyses are already producing robust evidence for addiction‐related practice and policy. As genetic variants associated with addictive behaviours are identified, the potential for Mendelian randomization analyses will grow. Methodological developments are also increasing its applicability.

## Introduction

Determining whether associations are causal is central to much addiction research but is challenging, with many observational associations unlikely to reflect causal relationships 1. Randomized controlled trials (RCTs), which support stronger causal inference, are not suited to all research questions—particularly as their external validity may be limited 2, 3, 4. Randomizing long‐term behaviours or environmental exposures in humans is unethical and impractical. Many causal questions, such as the long‐term consequences of consuming potentially harmful, addictive substances, cannot be answered with RCTs.

Mendelian randomization (MR) provides a tool for assessing the causal effects of behaviours on outcomes, although only when genetic variants associated with behaviours are known 5, 6, 7, 8. While previous reviews of MR exist 9, here we provide an up‐to‐date general introduction targeted specifically at addiction researchers. We note that other approaches to causal inference using observational data exist (including natural experiment approaches and statistical techniques such as propensity score‐matching, time–series analysis and structural equation modelling) 10, 11. We start by revisiting challenges to causal inference in traditional observational studies, explain how MR studies potentially overcome them and outline challenges and possible solutions when applying MR. Throughout, we illustrate MR's principles with two case studies: tobacco smoking as a possible cause of mental health problems (Box 2) and alcohol consumption as a possible cause of cardiovascular disease (Box 3). We conclude with some emerging methodological developments.

## Challenges to causal inference

Traditional observational studies face three major threats to establishing whether or not an association is causal 12.

First, characteristics of people with addictive behaviours (e.g. those who smoke or drink excessively) may differ systematically from other people. In naive comparisons of exposed and unexposed groups, these confounding factors are often responsible for observed differences in outcomes. Theoretically, if all confounding factors were measured and accounted for perfectly, an observational study could establish the effect of a behaviour accurately (provided other biases did not exist) 13. However, in practice it is difficult or impossible to identify all potential confounders. Furthermore, adequate control of confounding during statistical analysis requires accurate measurement of confounders, with even modest measurement error resulting in bias 14.

Secondly, it can be difficult to establish the direction of causation (i.e. whether reverse causation exists). While longitudinal studies may help (and are therefore more useful in causal inference), this is not always the case; the timing of the outcome in relation to behaviour may be uncertain. For example, when examining alcohol consumption and heart disease (Box 2) it is possible that behaviour change occurred before the early stages of disease were detected and diagnosed.

**Figure 1:**
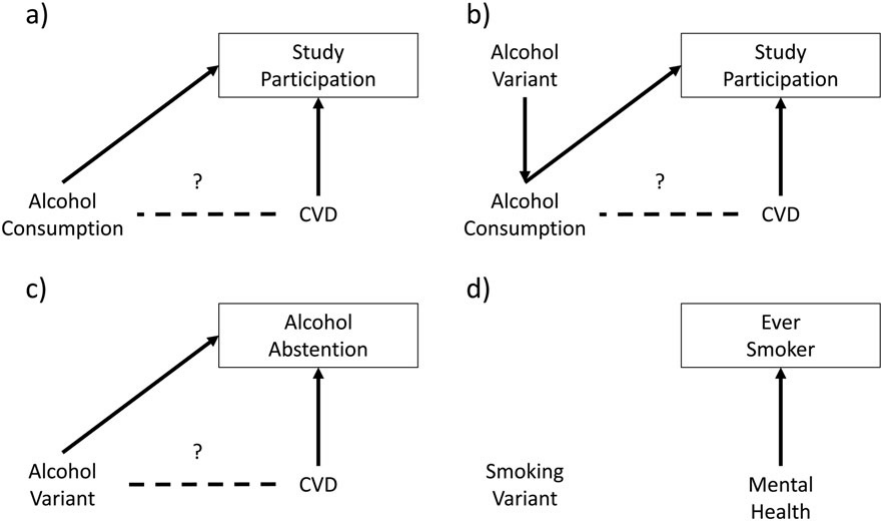
Directed acyclic graphs illustrating collider bias. (a) Collider bias within a traditional observational study arising from sample selection. The box around study participation indicates stratification on this variable. As study participation is influenced by both alcohol consumption and cardiovascular disease (CVD), stratification induces an association between these variables, indicated by the dashed line. (b) Mendelian randomization study which is still subject to collider bias. (c) Abstention is influenced by the genetic variant and the health outcome, inducing collider bias when stratifying by abstention. (d) The genetic variant is not associated with whether someone ever becomes a smoker, so there is no collider bias when stratifying on smoking status

**Figure 2:**
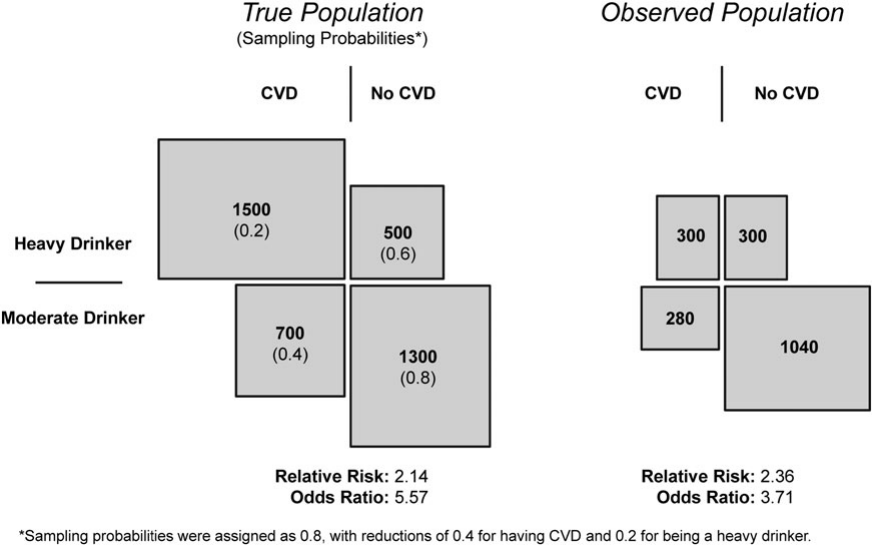
An illustration of collider bias arising from sample selection in a hypothetical study investigating the effect of alcohol consumption on cardiovascular disease (CVD). The left‐hand side of the figure demonstrates the relationship between a binary measure of drinking status and cardiovascular disease (based on fictitious data). Numbers in brackets indicate the probability of being recruited into the observed study population on the right‐hand side. Differences between the relative risks and odds ratios illustrate the collider bias arising from the selection process

Thirdly, collider bias may occur, whereby stratification on a common effect can result in a spurious correlation between otherwise independent variables 12. Figure 1a illustrates this principle (Fig. 1b–1d illustrates collider bias in the context of MR, and is discussed later). This threat to causal inference is perhaps less intuitive than either of the above, but can impact upon the strength and direction of associations observed 12, 15, 16. For example, moderate alcohol drinkers are more likely to participate in research 17, 18, 19. If those without cardiovascular disease (CVD) are also more likely to participate, estimates of association between alcohol consumption and CVD in observed populations will be biased. Figure 2 demonstrates this with simple, hypothetical data. Limiting the analysis to those who participate constitutes conditioning on a common effect and may induce bias 20: if an individual in the study sample is a heavy drinker, then they will be less likely to have CVD. Conditioning on other variables (e.g. as a result of stratification or statistical adjustment) can, similarly, result in bias.

## Principles of Mendelian randomization

In MR, genetic variants are used as proxies for the exposure of interest, which helps to avoid some of the problems described above. MR is an example of instrumental variable (IV) analysis that has long been used by economists to study causal effects 21. Randomization in an experimental study might be considered the purest form of instrumental variable 22. An instrumental variable is a proxy for the exposure of interest. While the correlation between instrument and proxy does not need to be strong, a poor correlation can be problematic and is referred to as a weak instrument (discussed later). The instrument should be unrelated to confounders and should impact the outcome only through its effect on the exposure. In an unbiased RCT, allocation by randomization is associated with the exposure group and is independent of confounders, so that the only pathway between allocation group and the outcome is through the exposure (treatment). An example of instrumental variable analysis using observational data from the economics literature is the use of minimum legal drinking age within US states as an instrument to study the effect of youth drinking on health and social outcomes 23. Assumptions underpinning this approach include observed and unobserved state characteristics that influence youth drinking being uncorrelated with their minimum legal drinking age policy, and that associations between the policy and outcomes operate only via youth drinking. Both these assumptions can be questioned (e.g. the assumption of the instrument being independent of confounders may be invalid if states that reduced legal drinking ages earlier experienced greater alcohol‐related harm previously).

In MR, genetic variants can be used as IVs to either assess whether a causal effect between exposure and outcome exists at all, or to measure the magnitude of the causal effect of the exposure on the outcome. Box 1 provides an overview of the principles for conducting a MR analysis. For a genetic variant to be a valid IV, it must satisfy three conditions:

1. The genetic variant must be associated with the exposure of interest (i.e. the behaviour being studied),
2. The genetic variant must be independent of any confounders of the exposure–outcome relationship being studied; and
3. The genetic variant should only affect the outcome through the exposure of interest.

**Box 1: Conducting a Mendelian randomization analysis**

The following is intended as a general guide to the principal stages of an MR analysis, acknowledging that this (and particularly step 7) may evolve as newer techniques emerge. Interested readers may wish to consider three other reviews 24, 25, 26. We present the examples in Boxes 2 and 3 structured around these steps so that the reader can see how the techniques are applied in practice.

1. *Define the research question, objectives and protocol*: define exposure(s) and outcome(s), data analysis methods, variables to be used, statistical power calculations 27, etc. This would typically include identification of a genetic variant that is known to be robustly associated with the exposure (or behaviour) of interest.
2. *Identify data source(s)*: identify potential data sources [e.g. published reports, summary results from genome‐wide association study (GWAS) consortia or individual‐level data] for the association between the genetic variant and the exposure, and for the association between the genetic variant and outcome. Two‐sample MR (see below) requires data for both associations to come from the different sources (see 68 for more details). In one sample MR, the gene exposure and gene outcome associations are estimated within the same sample.
3. *Estimate the gene–exposure association*: (i.e. test condition 1) for example, by regression of the exposure variable on the effect allele (or genetic risk score if multiple genetic variants are in use). If possible, calculate a partial *F*‐statistic, which provides an indication of the strength of the genetic instrument 28.
4. *Estimate associations between the genetic variant and measured confounders*: (i.e. a falsification test of condition 2) again, this might take the form of regression of measured confounders on the effect allele or genetic risk score. If multiple study populations are in use, do this for all populations where data on measured confounders are available.
5. *Estimate the gene–outcome association*: for example, by regression of the outcome on the effect allele or genetic risk score, this is often referred to as the ‘reduced form’ 29, 30.
6. *Estimate the magnitude of the causal effect*: this step is not necessary if the researcher is interested only in whether a causal effect exists, but if there is interest in the magnitude of effect this can be estimated as the ratio of the gene–outcome (reduced form) to the association between the exposure (or behaviour) and the genetic variant. Alternatively, one can first estimate the relationship between the genetic variant and the behaviour—for example, using conventional regression methods—and then estimate the relationship between the predicted behaviour from the first regression model and the outcome. In practice, rather than estimating two separate models, a jointly estimated two‐stage regression model may be used to take account of uncertainty in the predicted values from the first stage. It is worth noting that the magnitude of the causal effect should not be calculated for certain genetic instruments (e.g. *CHRNA5‐A3‐B4* which is used in Box 2 cannot be used to assess the magnitude of the causal effect for cigarettes per day on lung cancer) 31.
7. *Assess the plausibility of assumptions*: consider whether in the study setting the results could have been affected by pleiotropy, canalization, population stratification or unmeasured confounding (see section on challenges with MR and strategies to overcome them). These issues should at least be discussed in reporting results or where possible, formally tested.

**Figure 3:**
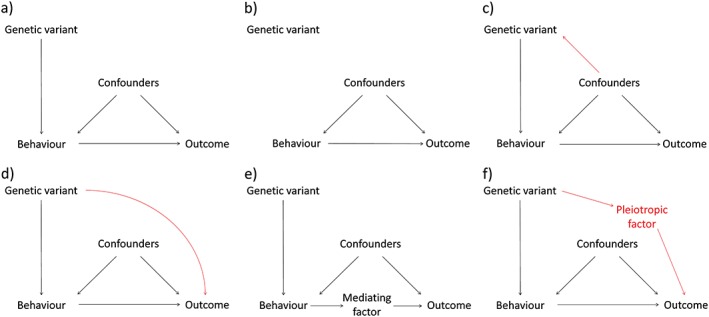
Directed acyclic graphs illustrating the assumptions underpinning valid Mendelian randomization (MR) studies. (a) All three assumptions for valid analysis are met. (b) No relationship between genetic variant and exposure, therefore assumption 1 is not met. (c) The genetic variant is not independent of confounders, therefore assumption 2 is not met. (d) The genetic variant does not exert its effect on the outcome only through the behaviour of interest, therefore assumption 3 (the ‘exclusion restriction’) is not met. (e) Mediated (or vertical) pleiotropy, where the behaviour of interest exerts its impact on the outcome via other intermediate factors. MR remains valid in this situation. (f) Biological (or horizontal) pleiotropy, where the genetic variant exerts effects on the outcome via both the behaviour of interest and via another pleiotropic factor. Note that this is an example of assumption 3 not being met. [Colour figure can be viewed at wileyonlinelibrary.com]

These conditions are illustrated graphically in Fig. 3. Figure 3a illustrates a scenario where all conditions have been met. Figure 3b–d shows violations of the three conditions.

Figure 3b shows violation of the first condition, that the genetic variant is associated with the behaviour of interest. Genetic variants often do not act as direct proxies. For example, the rs1051730 variant in the *CHRNA5‐A3‐B4* nicotinic receptor subunit gene cluster is associated with the heaviness of smoking in smokers, rather than smoking uptake per se (see Box 2 for an example using this variant for MR). It is therefore important to consider what specific aspect of behaviour the genetic variant reflects when interpreting MR results.

**Box 2: Cigarette smoking and mental health**

*Define the research question, objectives and protocol*: prevalence of smoking is higher among individuals with psychiatric disease (e.g. depression, schizophrenia) than in the general population, and individuals with these conditions tend to smoke more heavily 32. However, it is unclear whether the relationship between smoking and mental health is causal and, if so, what the direction of causality is. Individuals may smoke in order to relieve the symptoms of psychiatric disease, but it is also possible that smoking could increase risk of psychiatric disease. Smoking heaviness was the exposure of interest and two mental health outcomes were considered: depression and schizophrenia, measured both as diagnoses and by medication use. The rs1051730 variant in the *CHRNA5‐A3‐B4* cluster of nicotinic receptor subunit genes is associated with increased smoking heaviness (number of cigarettes smoked per day) among smokers, so was considered a potential instrumental variable. Associations were estimated using traditional regression.
*Identify data source*: individual‐level data from 63 296 participants in the Copenhagen General Population Study (CPGS).
*Estimate the gene–exposure association*: in the CPGS study, ever smokers with 0.1 and 2 risk (T) alleles smoked an average of 13.6, 14.5 and 15.6 cigarettes per day, respectively (*P*‐value = 1 × 10^−47^).
*Estimate associations between the genetic variant and measured confounders*: there was no clear evidence that the rs1051730 risk variant was associated with age, sex, education, marital status, income, alcohol consumption or physical activity.
*Estimate the gene–outcome association*: among ever smokers, the smoking heaviness increasing (T) allele of rs1051730 was associated with increased odds of antipsychotic medication use [odds ratio (OR) for TT homozygote compared to CC homozygote: 1.16 (95% confidence interval (CI) = 1.02–1.31]. Due to the nature of the allele used for the MR analysis, it is not possible to estimate a useful causal effect estimate for the number of cigarettes per day on the outcome. A similar trend was observed for schizophrenia diagnoses, although statistical evidence for this association was weak. There was little evidence that this variant was associated with depression or antidepressant medication use among ever smokers.
*Assess the plausibility of assumptions*: the rs1051730 variant is associated closely with the *CHRNA5* nicotinic receptor subunit gene, which has been shown to alter response to nicotine and subsequently affects how much tobacco is consumed among smokers. Therefore, there is a plausible biological mechanism linking this variant with smoking behaviour. It is believed not to influence the likelihood of someone becoming a smoker in the first place (i.e. Fig. 1d), so there is little risk of collider bias when stratifying by smoking status. Analyses stratified by smoking status showed that among never smokers the rs1051730 T allele was not associated with antipsychotic medication use (OR for TT homozygote compared to CC homozygote: 1.07 (95% CI = 0.87–1.31), which provides some evidence against pleiotropy because rs1051730 cannot be associated with heaviness of smoking in never smoking individuals. However, this effect estimate was also not clearly different from the effect estimate among smokers, so the results should be treated with some caution.

The second condition ensures that the genetic variant is not related to confounders (violated in Fig. 3c). While condition 1 can be tested empirically, condition 2 can, at best, only be tested partially. Researchers may demonstrate statistical independence of the genetic variant from measured confounders (for example in Box 2 the rs1051730 variant was not associated with several measured confounders). However, a theoretically informed argument for why an instrument should be independent of other unmeasured confounders is also needed 33. There are good reasons to expect this with genetic variants. According to Mendel's first law, each of the parent's two copies of a given section of DNA has an equal chance of being inherited, with the environment not influencing which copy produces a viable fertilized egg 34. The second law states that the two copies of a gene are inherited approximately independently from each other and from other genetic variants. The most common version of a specific genetic variant is referred to as the major allele and the least common is called the minor allele. These two laws in combination imply that any specific allele should be distributed randomly across the population, provided it has been transmitted stably across several generations and people's choice of partner is not influenced by the allele (i.e. there is no assortative mating).

Within econometrics, condition 3 is referred to as the ‘exclusion restriction’. This means that the causal pathway between the genetic instrument and the outcome occurs only through the behaviour of interest. It would be violated if the genetic variant had an impact upon the outcome through factors other than the exposure of interest—i.e. the genetic variant was acting as a proxy for other factors as well as the behaviour being studied (see Fig. 3d). Condition 3 also cannot be tested directly, but indirect supportive evidence can help in its evaluation (discussed later).

When a genetic variant is a valid IV 35, MR can overcome the first two of the three threats to causal inference outlined above. First, with regard to confounding, genetic variants are expected to be distributed approximately randomly across the population, and should therefore be independent of confounders of exposure–outcome relationships 1. Secondly, genotype is determined at conception and germline DNA is not modified thereafter, precluding reverse causation. With respect to the third threat, it has been suggested that selection bias may be less problematic in MR studies than in observational studies; for example, the distribution of genetic variants was found to be similar in blood donors (a highly selected group) compared with the UK general population 36, 37. However, collider bias may still be induced if both the behaviour and outcome of interest are related to study participation (see Fig. 1b). Even weak selection biases may influence outcomes of MR studies 20, as small biases in estimated gene–outcome associations can result in large changes to the causal behavioural–outcome estimates 38, 39. Thus, compared to conventional observational research, MR overcomes challenges of confounding and reverse causation, but selection bias may remain problematic. MR approaches can also be used to investigate whether behaviours impact upon prognosis after disease diagnosis—for example, while smoking causes lung cancer it is not clear whether it also influences prognosis 40. MR can therefore be used to understand the effects of addictive behaviours on disease prognosis, which may differ from their effects on aetiology, in turn informing clinical advice given to patients who have received a diagnosis. However, data sources for MR of progression are currently more limited than for studying aetiology.

## Challenges with Mendelian randomization and strategies to overcome them

We now describe some of the most important challenges with MR and commonly used strategies for addressing them. At the outset, we note that the need to have genetic data available is an important potential limitation—if DNA has not already been collected it may not be feasible to do so.

First, the genetic variant should be distributed randomly across the sample being analysed. However, the variant's distribution may differ between historically separate human populations, even though it is distributed randomly within each population 41. If outcome risks also differ between such populations, then the effect estimate may be confounded—a phenomenon referred to as ‘population stratification’. Restricting analysis to a single ethnic group (e.g. analysis of European ancestry individuals only) or statistical adjustment for ancestral information can reduce this risk.

Secondly, a crucial challenge is finding an appropriate genetic instrument, typically from a GWAS of the exposure of interest 42. These studies compare the frequency of genetic variants throughout the human genome in people who exhibit a behaviour with those who do not 43, 44. GWAS, which typically combine large sample size, statistical stringency (to account for the large number of statistical tests) and replication in an independent sample, have a good track record in identifying genetic variants reliably (i.e. alleles) associated with behaviours. The genetic variant need not be related causally to the behaviour, as long as they are associated reliably, so MR does not require knowledge about the function of the genetic variant. Nevertheless, causal inference is strengthened with understanding of the biological process. Without understanding how a gene exerts its function, we can be less certain that any effects are caused genuinely by the behaviour (i.e. assumption 3; see discussion of pleiotropy below).

A third challenge is that very large sample sizes are needed in MR because individual genetic variants typically exert small influences on behaviour and exposure measurement is often poor 45. Thus, studies may have low statistical power, especially for estimating the magnitude of causal effects, which requires estimation of two associations. Without large sample sizes, weakly associated genetic variants may yield estimates biased towards the naive observational association 46, 47, 48. To counter this, several genetic variants that are all associated with a behaviour may be combined to create a polygenic risk score 49. This could simply be a count of genetic variants that increase the behaviour, but more refined approaches weight the score so that more predictive genetic variants are weighted more strongly 50. While polygenic risk scores help address the weak instrument problem, the score must satisfy the three conditions for a valid instrument and may not do so if individual components do not themselves satisfy the three conditions 32.

An additional approach that makes achieving large sample sizes easier is two‐sample MR 51. As noted earlier, two associations are estimated typically when assessing the magnitude of a causal effect in MR: the association between genetic variant and behaviour and the association between genetic variant and outcome. In two‐sample MR these two associations are estimated from different samples, which is unproblematic provided that the underlying population from which the samples are drawn are the same. This reduces the need to access individual participant data.

A fourth challenge is especially problematic when assessing the magnitude (rather than presence) of a causal effect. As noted earlier, condition 3 (the exclusion restriction) requires no causal pathway between the genetic variant and the outcome, except through the exposure of interest. In MR studies, pleiotropy can violate this assumption 52. Two forms of pleiotropy can be distinguished 53, 54. Mediated (or vertical) pleiotropy occurs when the genetic variant is associated with a factor on the pathway between the behaviour and outcome, but only because of its effect on the behaviour (see Fig. 3e). This does not violate the exclusion restriction, as it is part of the pathway through which the behaviour exerts an effect. In contrast, biological (or horizontal) pleiotropy occurs when the genetic variant impacts upon a different biological pathway unrelated to the behaviour of interest, and therefore violates condition 3 (Fig. 3f). Methods to investigate, and to some extent relax, this assumption are described later.

Finally, cautious interpretation is necessary, bearing in mind the nature of the causal effect being estimated. For example, the study in Box 3 provides an estimate of the effect of the rs1229984 genetic variant on heart disease, indicating a causal effect of alcohol consumption. However, this genetic variant was associated both with units of alcohol consumed per week and with binge drinking. Attempts to estimate the magnitude of causal effects for specific drinking behaviour patterns, such as units per week, or binge drinking would be biased due to violation of condition 3 (as the genetic variant affects both behaviour patterns). Even when a genetic variant meets the three conditions to be a valid instrument, it may not provide an unbiased estimate of the effect of the specific aspect of behaviour that is of interest or has been measured 55. Also, as genetic variants are established at conception, the estimated causal effect is of a life‐long tendency towards a certain behaviour (or susceptibility to its impact) 56, 57. Furthermore, physiological adaptations may reduce a gene's effects (referred to as canalization) 58, while a gene's effects may be observed only under specific environments or exert impacts only at specific points during the life course (i.e. critical periods).

**Box 3: Alcohol consumption and coronary heart disease**

*Define the research question, objectives and protocol*: observational studies often report a J‐shaped association between alcohol and cardiovascular outcomes such as coronary heart disease 59. Risk is lowest for light to moderate drinkers, but increases for non‐drinkers and for heavier or more hazardous drinkers. Increased risk among heavy drinkers and abstainers may be due to reverse causation (e.g. abstainers may not drink because they have poorer health), selection bias or confounding from other social, life‐style or health factors. Alcohol consumption, measured in weekly units (where 1 unit = 4 g ethanol), was the main exposure of interest. The primary outcome considered was coronary heart disease. The minor allele at rs1229984 of the alcohol dehydrogenase 1b gene (*ADH1B*) has been identified in previous research as being associated with lower alcohol consumption and so was identified as a potential genetic instrument. Associations were estimated with standard regression techniques in multiple studies and then pooled using meta‐analysis.
*Identify data sources*: individual participant data on 261 991 participants of European ancestry were gathered from across 56 genetic studies.
*Estimate the gene–exposure association*: carriers of the risk allele at rs1229984 consumed fewer units of alcohol per week (17.2% fewer on average; 95% CI = 18.9–15.6%), had lower odds of heavy drinking (OR = 0.70; 95% CI = 0.68–0.73) or binge drinking (OR = 0.78; 95% CI = 0.73–0.84) and higher odds of abstention (OR = 1.27; 95% CI = 1.21–1.34) compared to non‐carriers.
*Estimate associations between the genetic variant and measured confounders*: the rs1229984 risk variant was not associated with physical activity or with most measures of smoking, although it was associated with slightly higher odds of ever smoking (OR = 1.06; 95% CI = 1.02–1.09), which is in the opposite direction to observational studies, and with slightly more years of education (0.04 standard deviations; 95% CI = 0.01–0.08).
*Estimate the gene–outcome association*: the rs1229984 risk variant was associated with reduced odds of heart disease among the whole sample (OR = 0.90; 95% CI = 0.84–0.96) and among drinkers (OR = 0.86; 95% CI = 0.78–0.94), suggesting that lower consumption is protective. Associations of the risk allele with heart disease did not differ further across light, moderate and heavy drinkers. This is contrary to the increased risk that would have been expected among light/moderate drinkers if moderate alcohol consumption had a true, causal protective effect relative to very low consumption. This suggests a linear, rather than a J‐shaped relationship between alcohol consumption and heart disease.
*Assess the plausibility of assumptions*: the *ADH1B* gene is involved in metabolizing alcohol, with the rs1229984 risk variant increasing the likelihood of unpleasant symptoms after consumption. Thus, there is a plausible biological mechanism connecting the genetic variant to alcohol consumption. The weak associations between the genetic variant and measured confounders are unlikely to explain any association between the genetic variant and heart disease, but it is possible that unmeasured confounders could be biasing results if transmission of the genetic variant across generations is related to alcohol consumption (i.e. assortative mating is occurring). Among non‐drinkers the rs1229984 risk variant was not associated with heart disease (OR = 0.98; 95% CI = 0.88–1.10), suggesting that it has no effect other than via reductions in alcohol consumption. This estimate may have been affected by collider bias because the genetic variant is associated with both abstention and heaviness of drinking among drinkers (see Fig. 1c), but there were no associations with measured confounders in stratified analyses, suggesting that these factors were unlikely to be biasing results.
*Implications for policy and practice*: light alcohol consumption does not protect from ischaemic heart disease, therefore efforts to reduce alcohol consumption are not likely to have any adverse impact on cardiovascular risk prevention.

## Assessing the robustness of Mendelian randomization studies

As noted above, biological pleiotrophic effects (violating assumption 3) threaten the validity of MR. While there remain no definitive ways of addressing this, several approaches now exist to explore potential bias. A theory‐informed approach is to make potentially informative comparisons to check the plausibility of assumption 3. For example, looking throughout countries with differing cultural norms for alcohol consumption can help to establish whether biological pleiotropy exists. In East Asian countries women tend not to consume alcohol, so genetic variants related to alcohol consumption would be expected to be associated with alcohol‐related disease outcomes in men but not in women 60. Cho and colleagues confirmed this by fitting a statistical interaction between the genetic variant and sex when carrying out a MR analysis using a South Korean sample, thereby providing further evidence that alcohol is related causally to an adverse cardiovascular risk profile 61. Similarly, in Box 3 a lack of an association between the genetic variant and heart disease among non‐drinkers increases confidence that the association among drinkers is due to drinking. Knowledge about the biological function by which the genetic variant exerts an effect is very helpful, as it provides greater confidence that the effect is via the behaviour rather than another mechanism 62. Box 2 illustrates how biological understanding can inform assessments of whether condition 3 is met in the case of a genetic variant associated with smoking.

Newly developed methods allow empirical investigation of MR assumptions. The funnel plot is used to identify small study bias in systematic reviews by looking for an association between study precision and effect size 63. In RCTs, increasing sample size should result in less variation in observed effect sizes (producing a symmetrical funnel‐shaped plot). The same principle has been applied to MR studies that make use of multiple genetic variants with differing strengths of association with the behaviour of interest 64. Stronger associations between genetic variants and the exposure should result in less variation and a symmetrical funnel plot. Asymmetry in the funnel plot indicates that the MR assumptions are not met (see Fig. 3). MR Egger regression builds on this to allow for overall biological (horizontal) pleiotropy across multiple genetic instruments to be estimated and the causal effect to be appropriately adjusted for. It can be applied when conducting two‐sample MR 65. An additional sensitivity analysis is to use a weighted median estimator, as the median estimate across several genetic instruments should be less prone to bias from confounders (i.e. violations of assumption 2), provided that a majority of the weighted analysis is based on valid instruments 66. The assumptions underpinning each of these techniques differ—therefore a consistent pattern of findings strengthens causal inference. However, these techniques require multiple genetic instruments acting as proxies for the same behaviour.

## Extensions to Mendelian randomization

Analytical tools for MR research are developing and being refined rapidly. Bidirectional MR is an extension of the traditional design utilizing genetic markers for different but inter‐related outcomes to investigate the direction and magnitude of the causal effects. For example, the causal relationship between cannabis use and schizophrenia remains controversial. Gage and colleagues studied two sets of genetic variants, one related to cannabis initiation and one related to schizophrenia risk, to understand more clearly the direction of causation 67. They found that schizophrenia‐related genetic variants were related strongly to cannabis initiation while genetic variants linked to cannabis initiation were associated weakly with schizophrenia. The authors therefore concluded that ‘cannabis initiation increases the risk of schizophrenia, but the size of the causal effect is small’ and there is ‘stronger evidence that schizophrenia risk predicts cannabis initiation’. The use of multiple genetic instruments to investigate the direction of causality can be extended to investigate multiple mediating factors 68. In such ‘network’ MR, genetic instruments for each mediating factor to be investigated are required, and the genetic instruments must be independent of each other 69.

Finally, factorial MR allows combinations of multiple behaviours to be investigated. Factorial MR is akin to a factorial RCT where the population is in effect allocated randomly to receive any combination of the behaviours under consideration 70. For example, the combination of excess alcohol consumption and obesity are known to result in far greater risk of liver cirrhosis than would be expected based on their additive effects (i.e. they show evidence of effect modification) 71, 72. However, observational studies of effect modification face threats to causal inference. Factorial MR therefore has similar advantages to a factorial RCT—allowing multiple behaviours to be investigated and compared against each other, singly or in combination. Again, availability of genetic instruments for the different behaviours and their biological independence are important considerations.

## Conclusions

MR adds to the range of study designs available to understand the causal effects of behaviours on outcomes of interest. It helps address key limitations of traditional observational studies, including confounding and reverse causation, but selection bias could remain problematic. MR studies potentially allow researchers to produce more robust evidence on questions of immense relevance to policy and practice. They can provide strong evidence of causation, subject to necessary assumptions which benefit from an understanding of the underpinning biological processes. However, two of the three assumptions underpinning MR cannot be tested definitively. Furthermore, genetic variants known to be associated with behaviours of interest are required and genetic data from a large number of people, including those exhibiting the behaviour of interest, are needed. A range of other causal approaches to observational research are available, with differing underpinning assumptions; their use in combination can be particularly powerful 10, 73. We have provided a broad overview of the topic so that interested readers are able to read critically and interpret findings from MR studies. The use of genetic instruments for gaining causal understanding is already yielding important insights into addiction research and will probably advance the field substantially in the future.

## Declaration of interests

None.
